# How Healthcare Provider Characteristics Affect Their Attitudes and Skills in Involving Families in Caring for Small and Sick Newborns Throughout the Perinatal Period

**DOI:** 10.1177/23779608251376225

**Published:** 2025-09-05

**Authors:** Christina Schuler, Alondra Ramos, George Edward Ntow, Riccardo E. Pfister, Faith Agbozo

**Affiliations:** 1 Institute of Global Health, Faculty of Medicine, 28535 University of Geneva, Geneva, Switzerland; 2 Institute of Nursing, School of Health Sciences, Zurich University of Applied Sciences (ZHAW), Winterthur, Switzerland; 3 The Dartmouth Institute for Health Policy and Clinical Practice, Geisel School of Medicine, Dartmouth College, Hanover, NH, USA; 4 Dodowa Health Research Centre, Dodowa, Ghana; 5 Neonatal and Pediatric Intensive Care Unit, University Hospitals of Geneva and Geneva University, Geneva, Switzerland; 6 Department of Family and Community Health, Fred N. Binka School of Public Health, 549574 University of Health and Allied Sciences, Ho, Ghana

**Keywords:** continuity of care, family-centered care, health personnel, Ghana, maternal and neonatal care, primary health care, regression analysis, self-perceived skills

## Abstract

**Introduction:**

Healthcare providers (HCPs) are essential in providing care and working with families with small and sick newborns throughout the perinatal care continuum. While clinical experience, education, and training influence HCPs’ attitudes and skills regarding family involvement in care, the specific factors affecting Ghanaian HCPs remain unclear.

**Objectives:**

To identify HCPs’ characteristics that influence their attitudes and self-perceived practice skills toward involving families in the perinatal care continuum for small and sick newborns.

**Methods:**

This analytical cross-sectional study involved 141 HCPs, including nurses, midwives, and medical staff, from one secondary and 13 primary healthcare facilities in Ghana. Participants completed an online survey using two standardized scales: (1) *“Family Importance in Nursing Care-Nursing Attitudes”* which assesses HCPs’ perceptions of the significance of family involvement in care and the (2) “*Family Nursing Practice Scale”* which evaluates the extent to which HCPs integrate family-systems approaches in their practice. Data analysis involved multiple linear regression models.

**Results:**

Self-perceived practice skills and attitudes towards family involvement influenced HCPs in multiple dimensions, including viewing “family as a burden” (associated with increased stress), “as a conversational partner” (as active participants in care), and as a “resource in care” (having their own coping resources). Availability of a family systems care work approach and prior education in family systems care significantly influenced HCPs’ self-perceived practice skills. HCPs skills and attitudes in family involvement influenced each other.

**Conclusion:**

Transforming attitudes towards family systems care across all levels, from hospital to home, should aim to improve the quality of newborn care. Tailored family systems care education, skills training, and institutional support are needed to enhance HCPs’ skills and attitudes across the perinatal care continuum for small and sick newborns.

## Introduction

Healthcare providers (HCPs) in hospitals and primary care, including nurses, midwives, and physicians, form the backbone of maternal and neonatal care services. They play an essential role in the prevention, diagnosis, and management of maternal and neonatal illnesses (WHO, 2024; Wirth, 2008).

Neonatal care in low- and middle-income countries (LMICs) comes with challenges, such as a lack of skilled human and physical resources, particularly for small and sick newborns (Bolan et al., 2021; Steenhoff et al., 2023). The vulnerability of these newborns, coupled with limited resources, underscores the need for effective healthcare (Steenhoff et al., 2023). Despite facing resource constraints, HCPs often exhibit resilience and ingenuity in delivering quality care, leveraging their skills and expertise to optimize outcomes for newborns and their families (Petrites et al., 2016).

The intersection of family dynamics and illness is a significant aspect of healthcare delivery (Wright & Bell, 2021). The functioning and coping strategies of families play a vital role in shaping how patients perceive and manage their experiences (Blöndal et al., 2014; Yang et al., 2022). Newborn development, growth, breastfeeding, and overall well-being are intricately linked to family dynamics, highlighting their influence in the early stages of life. Family involvement in care also decreases length of hospital stay, parental stress, and anxiety (North et al., 2022). More positive outcomes are reported from families engaged throughout the care continuum, starting from pregnancy (Nyasulu et al., 2025). This continuum should address not only immediate medical needs but also emotional, social, and psychological aspects of the patient and his family (Khatri et al., 2023; Nyasulu et al., 2025; WHO, 2025).

One way to address these affective aspects is through family systems care (FSC). FSC is a systemic approach where the family is considered the unit of care, and where emotional and psychosocial support is provided through family involvement (Petursdottir et al., 2019). Family involvement, as shown by models like the Calgary Family Assessment Model (CFAM) and the Calgary Family Intervention Model (CFIM), enhances the quality of care and contributes to a more positive experience for mothers, newborns, and their families throughout the care continuum (Abukari & Schmollgruber, 2023).

The CFAM helps HCPs to evaluate family structure and development by identifying family responses to health issues, including strengths, resources, challenges, and the impact of illness. The CFIM guides the selection of tailored interventions to improve family functioning across cognitive, emotional, and behavioral domains. It employs targeted family care interventions based on interventive questions and commendations (Comments on family or individual strengths, competencies, and resources during conversations) (Shajani & Snell, 2023).

## Review of Literature

Attitudes and skills of HCPs toward family involvement in care are shaped by a combination of factors (Imanipour & Kiwanuka, 2020). Extensive clinical experience, advanced education, and relevant training are more likely associated with positive attitudes and effective family involvement (Blöndal et al., 2014; Hagedoorn et al., 2021; Østergaard et al., 2020). Personal experience with illness and work in institutions with a clear family systems structure are also associated with more positive attitudes (Hoplock et al., 2019; Hsiao & Tsai, 2015; Østergaard et al., 2020). However, a few studies have reported inconsistencies in attitudes and skills, suggesting that the influence of education level, workplace philosophy, and experience may be of lesser importance (Hagedoorn et al., 2021; Zwicky et al., 2022). Understanding the characteristics that empower HCPs is essential for developing targeted interventions to achieve effective FSC (Benzein et al., 2008b; Zwicky et al., 2022).

Despite the central role of HCPs in maternal and neonatal care, there is a lack of research examining how their characteristics influence attitudes and skills in FSC in the African context (Chironda et al., 2022; Kiwanuka et al., 2023). Evidence from high-income countries has explored the influence of HCPs’ characteristics on nurses’ attitudes about the importance of involving families in care. These studies explored characteristics such as education level, gender, years of work experience, and personal experience with illness in the family, though always within a single setting (Hagedoorn et al., 2021; Linnarsson et al., 2015; Luttik et al., 2017; Shamali et al., 2022; Svavarsdottir et al., 2018). To our knowledge, no study has investigated how HCPs’ characteristics influence perceived practice skills and attitudes across the maternal and newborn care continuum.

Therefore, there is clearly a need for research to clarify the influence of HCPs’ characteristics on attitudes and skills, even more so in the African healthcare settings, where such studies are exceedingly rare.

This study sought to understand how HCPs’ characteristics influence perceived practice skills and attitudes toward family involvement in care for small and sick newborns throughout the perinatal care continuum.

## Methods

### Study Design

This study was an analytical cross-sectional online survey conducted among HCPs from June to August 2023. The survey adhered to the Strengthening the Reporting of Observational Studies in Epidemiology guidelines (STROBE-Initiative, 2024) for articles reporting cross-sectional studies.

### Setting

Hohoe is the second-largest municipality in the Volta Region of Ghana. Compared with other municipalities in the region, Hohoe has an appreciable range of healthcare facilities that provide services spanning from newborn to adult healthcare. The municipality has eight health centers and five community-based health planning and services (CHPS) facilities that provide basic care at the primary level of the health system (community level), mainly maternal, child, and neonatal care. The regional hospital provides some of these essential services: antenatal care, basic and emergency obstetric and essential newborn care, as well as post-surgical care for women who have undergone cesarean sections. Small or sick newborns receive special care in the neonatal intensive care unit (NICU) and the public health and nutrition unit and a child welfare clinic providing growth monitoring, nutrition counseling, and vaccination. Various categories of HCPs, including specialist pediatric nurses, general nurses, community health nurses, midwives, obstetricians or gynecologists, pediatricians, general physicians, and physician assistants, render the services mentioned above.

The inhabitants are engaged in varied trade and economic activities, with the informal sector being the largest employment provider, entailing primarily crop and livestock farming, petty trading, and small-scale artisanal work. Only a small proportion of the population is employed in the formal sector (Ghana Health Service, 2023; Ghana Statistical Service, 2019; Ministry of Food and Agriculture, 2023).

### Participants and Eligibility Criteria

This survey comprised nurses, midwives, and medical staff working with pregnant or laboring women and/or small/sick newborns and their families. The study participants worked either at the antenatal care clinic, labor ward, NICU, postnatal ward, public health, and nutrition unit, or at one of the 13 primary care facilities.

Students undergoing basic vocational training and internship in healthcare were excluded as the aim was to capture exclusively the experience of HCPs who had at least completed basic professional training.

### Sample and Sample Size

At the time of data collection, 208 HCPs were involved in maternal and newborn care across various units in the hospital and at the primary care level. Given the total number of eligible HCPs, a census sampling approach was opted for and all eligible HCPs were invited to participate in the study, following the recommendation of Israeli (1992). Of the 208 HCPs invited to take part, 143 HCPs responded, yielding a response rate of 68.8%. The current analysis included 141 HCPs*:* 60.3% (n = 85) were nurses, 36.2% (n = 51) midwives, and 3.5% (n = 5) medical staff. Two participants were excluded because they were the sole respondents from their hospital ward, preventing meaningful ward-level analysis.

### Data Sources/Measurement

Data collection was based on four different standardized questionnaires. Here, findings are reported on the Family Importance in Nursing Care-Nursing Attitudes (FINC-NA) scale (Benzein et al., 2008a), which measures HCPs’ attitude towards the importance of family involvement in care, and the Family Nursing Practice Scale (FNPS), which assesses HCPs family care practices and the family-healthcare provider relationship (Simpson & Tarrant, 2006). The FNPS is freely accessible online; however, permission to use the FINC-NA was obtained from its authors. Although originally created for nurses, these scales are increasingly used among other HCPs like midwives (Naef et al., 2020a) and physicians (Naef et al., 2020b). The scales assessing contextual analysis and implementation outcomes are addressed in distinct publications.

Acknowledging the bidirectional nature of HCPs’ skills, attitudes, and perceptions of family involvement, the FNPS, and FINC-NA scales were included as either predictors or outcomes, depending on the specific aim of each model. No single model included the same variable as both predictor and outcome, and this distinction is indicated in each model.

### Healthcare Provider Attitudes Towards Family Involvement

The original English version of the FINC-NA tool was used to assess attitudes of HCPs towards the importance of family involvement in healthcare. Responses were gauged on a Likert scale ranging from 1 (strongly disagree) to 5 (strongly agree). The total score ranges from 26 (minimum) to 130 (maximum), where higher scores reveal a more positive and open attitude toward family involvement (Benzein et al., 2008a). The 26 items are captured and categorized under four sub-scales. Subscale 1, “Family as a Resource in Nursing Care” (Fam-RNC), assesses positive attitudes towards families and their healthcare involvement (10 items, 10–50 score). Subscale 2 represents “Family as a Conversational Partner” (Fam-CP), which assesses whether HCPs actively involve family members in care activities (8 items, score range: 8–40). Subscale 3 represents “Family as a Burden” (Fam-B) and measures HCPs stress levels in response to family presence (4 items, score range: 4–20). Subscale 4 represents “Family as Their Own Resources” (Fam-OR), exploring whether families are considered having their own resources for coping (4 items, score range: 4–20). The psychometric properties of the tool have been evaluated by Benzein et al. (2008a) with an internal consistency of α = 0.88.

### Healthcare Provider Practice Skills in Working With Families

The original English FNPS tool was used to measure HCP appraisal of practice skills and their reflection on working with families. The tool comprises ten items on a 5-point Likert scale, ranging from one (high level) to five (low level) (Benzein, 2008a). Lower scores indicate a more positive assessment of family care skills, with total scores ranging from 10 (minimum) to 50 (maximum). The FNPS tool has an internal consistency of α 0.86 (Simpson & Tarrant, 2006).

### Healthcare Provider Characteristics

The independent variables of interest were sociodemographic characteristics. Sociodemographic data of HCPs, such as highest academic degree, years of work experience, experience with illness in their own family, prior education in FSC, as well as the FSC approach implemented at the workplace were collected. The nature of these variables (categorical or continuous) informed the choice of the regression analysis run.

### Procedure

Prior to the study, the instrument was pilot-tested in a nearby municipality. The process yielded feedback regarding the questionnaire's length and the sociodemographic variables involved. Comparable to a study from Uganda (Imanipour & Kiwanuka, 2020), using the same scales, there were no issues with clarity or appropriateness*.* The questionnaire was administered using Kobo Collect (2023), an open-source software for online surveys. Hospital and district health management teams provided contact information for supervisors of the targeted departments. These supervisors then distributed the questionnaire link to eligible participants through official departmental WhatsApp groups used for professional communication.

Responses were downloaded into Microsoft Excel, cleaned, and exported into Stata version 17 for analysis.

### Data Analysis

Descriptive statistics were used to summarize the sociodemographic characteristics of the study participants and responses obtained from the FINC-NA and FNPS questionnaires. The Gauss–Markov Theorem was used to verify assumptions. Multicollinearity was assessed using the Tolerance Index, which calculated tolerance as 1/VIF (Variance Inflation Factor) (Kim, 2019). A tolerance index below 0.1–0.2 indicated multicollinearity. Multivariate linear regression analyses were performed to identify the individual contribution of the demographic, organizational, and professional factors to the FNPS total score, FINC-NA total score, and subscale scores. Each of the total FNPS and FINC-NA scores, as well as the four FINC-NA sub-scores, were used as a dependent variable in separate models. Based on this, six multivariate linear regression models were fitted. A significance level of α < 0.05 and a 95% confidence interval (CI) were used for all statistical analyzes.

### Ethical Considerations

This study received ethical approval from the Ghana Health Service Ethical Review Committee (GHS-ERC 027/03/23). Permission to conduct the study was gained from the Volta Regional Hospital Management and the Hohoe Municipal Health Directorate. The Declaration of Helsinki guided this study approach (World Medical Association, 2024). Study information was provided via the online link as requested by the ethics committee, and participants consented online prior to participation. The written consent was recorded and stored securely. Data collection and analysis were done anonymously.

## Results

### Sociodemographic Characteristics and Professional Experience of Healthcare Providers

Of the 141 HCPs, 80.1% were female, with 67.7% falling between the ages of 30 and 39 years. Almost half (48.2%) held a diploma, while 33.4% held a certificate. Also, 59.6% reported no training in FSC, but 62.4% testified to the availability of a general approach to family care at their workplace. Additionally, 77.3% had personally experienced a family member with a serious illness. Detailed characteristics are displayed in Table 1.

**Table 1.** Sociodemographic Characteristics and Professional Experience of the Healthcare Providers.

Characteristics	Categories	N = 141
		n (%)
Age (in years)	(Mean/SD)	33 (±5.0)
	20–29	36 (25.5)
	30–39	94 (67.7)
	40+	11 (6.8)
Gender	Female	113 (80.1)
Cadre of healthcare providers	Midwives	51 (36.2)
	Pediatric nurses	3 (2.1)
	General nurses	28 (19.9)
	Enrolled nurses	16 (11.3)
	Community health nurses	38 (27.0)
	Medical staff^a^	5 (3.5)
Educational level	Certificate	47 (33.4)
	Diploma	68 (48.2)
	Bachelor	20 (14.2)
	Master	3 (2.1)
	Other^b^	3 (2.1)
Workplace unit	Antenatal care unit	10 (7.1)
	Labor ward	17 (12.1)
	Neonatal intensive care unit	17 (12.1)
	Postnatal care unit	9 (6.4)
	Public health and nutrition unit	8 (5.7)
	Health centers	54 (38.2)
	Community health planning services	26 (18.4)
Work experience in the unit (in years)	(Means/SD)	5 (±3.7)
	0–3	99 (70.2)
	4–9	37 (26.3)
	10+	5 (3.5)
Prior education in family systems care	Yes	58 (40.6)
	No	85 (59.6
Family systems care used at workplace	Yes	88 (62.4)
	No	53 (37.6
History of family member with serious illness	Yes	109 (77.3)
	No	32 (22.7)

a Medical staff includes: 2 general physicians, 2 physician assistants, 1 medical assistant.

b Educational Level: Other includes other postgraduate educations such as a specialization in medical or nursing care.

### Factors Predicting Health Providers’ Perceptions of the Family as a Resource in Nursing Care

This first regression model assessed the factors influencing HCPs’ perceptions of families as a resource in nursing care (Fam-RNC) (Table 2). The skills (FNPS) of the HCP was the only factor significantly associated with the Fam-RNC subscale, with a negative coefficient of −0.257. Higher levels of skills (indicated by a lower FNPS score) were associated with a stronger perception of families as a resource in care. The model explained 24.8% of the variance in the Fam-RNC subscale (R-squared). The overall model was statistically significant, as indicated by the F-test.

**Table 2.** Multiple Linear Regression Model Showing How Much Family as a Resource in Nursing Care (Fam-RNC) is Affected by the Sociodemographic Characteristics and Professional Experience of the Healthcare Providers.

	Coefficient	Standard error	*p*-value	95% Confidence interval
Age	.09	.15	.57	[−.208, 0.379]
Gender (ref: Male)	−1.27	1.47	.39	[−4.17, 1.63]
Educational level (ref: diploma)				
Certificate	−1.99	1.39	.16	[−4.74, .76]
Bachelor	−2.24	1.73	.20	[−5.67, 1.19]
Master	3.33	4.16	.43	[−4.90, 11.56]
Other	1.29	3.97	.75	[−6.58, 9.16]
Healthcare unit (ref: CHPS^b^)				
Antenatal care unit	−1.26	2.82	.66	[−6.85, 4.33]
Labor Ward	−.82	2.43	.73	[−5.63, 3.98]
Neonatal intensive care unit	2.15	2.16	.32	[−2.13, 6.42]
Postnatal care unit	−1.33	2.62	.61	[−6.52, 3.86]
Public health and nutrition unit	.36	2.67	.89	[−4.92, 5.64]
Health center	2.04	1.60	.20	[−1.12, 5.2]
Healthcare provider skills (FNPS)^a^	−.26	.08	0***	[−.41, −.11]
Prior training in family systems care (ref: No)	.56	1.13	.62	[−1.68, 2.81]
Family systems approach at workplace (ref: No)	1.27	1.17	.28	[−1.05, 3.60]
Work experience in the unit (years)	.03	.20	.87	[−.36, .43]
Previous illness (ref: No)				
Yes	1.04	1.37	.45	[−1.70, 3.74]
Constant	41.23	5.80	0***	[29.75, 52.71]
Mean dependent variable	38.03	SD dependent variable		6.76
R-squared	0.25	Number of observations		141
F-test	2.39	Prob > F		0
Akaike crit. (AIC)	933.98	Bayesian crit. (BIC)		987.05

Dependent variable: Subscale: Family as a Resource in Nursing Care (Fam-RNC).

a FNPS (Family Nursing Practice Scale): Total score ranging from 50 = low skill to 10 = high skill.

b CHPS (Community-based Health Planning and Services).

*** *p* < .01.

### Factors Influencing Healthcare Providers' Perception of Family as a Burden

This second regression model explored factors influencing HCPs’ perception of families as a burden (Fam-B subscale of the FINC-NA) (Table 3). Working in the public health and nutrition unit, as well as the skills of healthcare providers (FNPS), had a positive and significant association with Fam-B. The model explained 24.7% of the variance in this subscale. HCPs working in the public health and nutrition unit were less likely to perceive families as a burden, whereas HCPs with weaker family nursing skills were more likely to view families as a burden. The F-test indicated that the model was statistically significant.

**Table 3.** Multiple Linear Regression Model Identifying Factors Influencing Healthcare Provider Perceptions of Families as a Burden (Fam-B).

	Coefficient	Standard error	*p*-value	95% Confidence interval
Age	.021	.06	.73	[−.10, 0.12]
Gender (ref: Male)	−.90	.59	.13	[−2.06, 0.27]
Educational level (ref: diploma)				
Certificate	.65	.56	.25	[−.46, 1.75]
Bachelor	−.45	.70	.52	[−1.83, .93]
Master	.44	1.67	.79	[−2.87, 3.75]
Other	.32	1.60	.84	[−2.84, 3.48]
Healthcare unit (ref: CHPS)^b^				
Antenatal care unit	1.15	1.14	.31	[−1.10, 3.40]
Labor ward	.30	.98	.76	[−1.63, 2.23]
Neonatal intensive care unit	−.026	.87	.98	[−1.74, 1.69]
Postnatal care unit	1.74	1.06	.10	[−.35, 3.83]
Public health and nutrition unit	4.03	1.07	0***	[1.91, 6.16]
Health center	.052	.64	.94	[−1.22, 1.32 ]
Healthcare provider skills (FNPS)^a^	.11	.03	0***	[.05, .17]
Prior education in family systems care (ref: No)	−.86	.46	.06*	[−1.76, .042]
Family systems care approach at workplace (ref: No)	−.46	.47	.33	[−1.39, .48]
Work experience in the unit (years)	.011	.08	.89	[−.15, .17]
Previous illness (ref: No)				
Yes	.079	.55	.89	[−1.01, 1.17]
Constant	7.14	2.33	0***	[2.52, 11.75]
Mean dependent variable	9.46	SD dependent variable		2.72
R-squared	0.25	Number of observations		141
F-test	2.37	Prob > F		0.00
Akaike crit. (AIC)	676.99	Bayesian crit. (BIC)		730.0

Dependent variable: Family as a Burden (Fam-OR) (FINC-NA subscale).

a FNPS (Family Nursing Practice Scale): Total score ranging from 50 = low skill to 10 = high skill.

b CHPS (Community-based Health Planning and Services).

*** *p* < .01, * *p* < .1.

### Factors That Determine the Practice Skills of Health Providers in Working With Families

The third regression model identified factors influencing HCPs’ skills in working with families, as measured by the FNPS scale (lower scores indicate higher skills), as the dependent variable (Table 4). HCP attitudes and the implementation of FSC in their work unit significantly influenced their skills (Table 4). The model explained 25.7% of the variance, and the overall model was statistically significant.

**Table 4.** Multiple Linear Regression Model for Factors Determining Practice Skills in Working With Families (FNPS Total Score).

	Coefficient	Standard error	*p*-value	[95% Confidence interval]
Age	−.11	.18	.55	[−.45, .24]
Gender (ref: Male)	−2.70	1.70	.12	[−6.12, .73]
Educational level (ref: diploma)				
Bachelor	−1.29	2.05	.53	[−5.36, 2.77]
Certificate	−1.04	1.64	.53	[−4.29, 2.21]
Master	−7.0	4.85	.15	[−16.61, 2.61]
Other (Other postgraduate education)	−3.96	4.66	.34	[−13.19, 5.27]
Healthcare Unit (ref: CHPS^b^)				
Antenatal care unit	.48	3.32	.89	[−6.10, 7.06]
Labor ward	2.52	2.85	.38	[−3.12, 8.15]
Neonatal intensive care unit	.26	2.56	.92	[−4.81, 5.32]
Postnatal care unit	−1.09	3.09	.73	[−7.19, 5.03]
Public health and nutrition unit	−4.68	3.13	.14	[−10.87, 1.52]
Health center	−2.4	1.88	.21	[−6.11, 1.33]
Healthcare provider attitudes (FINC-NA)^a^	−.11	.05	.02**	[−.21, −.018]
Prior education in family systems care (ref: No)	.74	1.34	.58	[−1.91, 3.38]
Family systems care approach at workplace (ref: No)	−4.30	1.33	0***	[−6.93, −1.67]
Work experience in the unit (years)	−.15	.23	.53	[−.61, .32]
Previous illness (ref: No)				
Yes	−1.30	1.61	.42	[−4.48, 1.88]
Constant	49.84	6.89	0***	[36.21, 63.47]
Mean dependent variable	28.91	SD dependent variables		8.01
R-squared	0.26	Number of observations		141
F-test	2.51	Prob > F		0.00
Akaike crit. (AIC)	979.81	Bayesian crit. (BIC)		1032.89

Dependent variable: Healthcare provider skills (Family Nursing Practice Scale - FNPS total score).

a FINC-NA (Family Importance in Nursing Care – Nursing Attitudes): Total scale ranging from 19 (less supportive attitude) to 95 (open attitude towards families).

b CHPS (Community-based Health Planning and Services).

*** *p* < .01, ** *p* < .05.

### Factors Influencing Healthcare Providers’ Perception of Families as Conversational Partners in Care

The fourth regression model examined factors influencing HCPs’ perceptions of families as conversational partners in care, with healthcare provider skills (FNPS) and other variables as independent factors (see Supplemental File 1). The dependent variable was the Family as a conversational partner (Fam-CP) subscale on the FINC-NA total scale. Healthcare provider skills (FNPS) had a statistically significant negative association with perceiving families as conversational partners in care (coefficient = −0.212, 95% CI = −0.341 to −0.082, *p* = .02) (see Supplemental File 1). The model explained 21.5% of the variance in the Fam-CP subscale. The F-test (*p* = .018) indicated that the model was statistically significant.

### Openness of Healthcare Providers to Recognize Families as a Resource

The fifth regression model examined factors influencing openness of HCPs to recognize families as a resource in care (Fam-OR subscale of the FINC-NA scale) (see Supplemental File 2). Previous education in FSC had a significant impact on Fam-OR (coefficient = 0.96, 95% CI = 0.03 to 1.89), indicating that individuals with such training were more receptive to viewing families as a valuable resource in their own care. The model explained 24.6% of the variance. The F-test (*p* = .02) confirmed the model's statistical significance.

### Factors Influencing Healthcare Providers' Attitudes Toward Family Involvement

In the sixth regression model, the FINC-NA total score was used as the dependent variable to examine factors influencing HCPs’ attitudes toward family involvement (see Supplemental File 3). Higher practice skills, indicated by lower FNPS scores (reversed scale), significantly predicted more positive attitudes (coefficient = −0.39, 95% CI = −0.71 to −0.06). The model explained 19.6% of the variance. The model was statistically significant (F-test, *p* = .04).

### Internal Reliability of the Scales

The internal consistency of the FINC-NA scale was confirmed by the Cronbach's alpha coefficients ranging from 0.63 to 0.79. For the FNPS tool, the Cronbach's alpha coefficients ranged from 0.71 to 0.73.

## Discussion

The attitudes of HCPs on family involvement in the care of small and sick newborns, notably self-perceived practice skills, were significantly associated with several health provider characteristics. They were also linked to the perception of the family as a burden, a conversational partner, and a resource in care. Availability of a FSC approach at the workplace and prior training in FSC accounted for most of the differences in family involvement and attitudes towards working with families. Public health and nutrition unit staff viewed families less as a burden, but other teams across the care continuum showed no statistically significant difference.

In contrast, previous studies identified variations in family involvement between healthcare teams, such as between neonatal and adult intensive care nurses, or pediatric and medical-surgical nurses (Cranley et al., 2022; Hetland et al., 2017; Zwicky et al., 2022). The differences identified may be attributed to high staff turnover in the investigated units (Schuler et al., 2023). Elevated staff turnover can lead to the depletion of critical competencies (Murless-Collins et al., 2025), including skills related to FSC. This may reduce HPCs’ confidence in delivering FSC, which can contribute to less favorable attitudes toward involving families in practice (Barreto et al., 2022). All other sociodemographic characteristics in this study were not statistically linked to attitudes towards family involvement.

Most prominently, higher HCPs’ self-perceived practice skills positively influenced their family involvement across multiple dimensions, including viewing the family as a conversational partner and as a resource in care, and less as a burden. These findings support evidence that emphasizes the role of skills in working with families, which significantly influences HCPs’ attitudes towards families (Hsiao & Tsai, 2015; Zwicky et al., 2022). HCPs with strong clinical confidence may indeed be more likely to express positive attitudes towards families, leading to better engagement and collaboration (Blöndal et al., 2014; Hagedoorn et al., 2021; Luttik et al., 2017). HCPs’ self-confidence is therefore likely to induce a virtuous cycle leading to enhanced patient satisfaction and improved health outcomes.

HCPs with prior FSC training showed increased awareness of families’ potential as their own resource in coping, consistent with previous findings (Barreto et al., 2022; Zwicky et al., 2022). Educational interventions emphasizing the importance of family in care promoted positive provider attitudes toward family involvement and fostered confidence in FSC (Broekema et al., 2018; van Oort et al., 2024). Kiwanuka et al. (2023) found that nurse assistants showed higher skills compared to nurses with higher education, potentially because of their closer interaction with families. In this study, however, all HCPs had approximately similar contact with families.

While studies demonstrate a positive impact of educational interventions, some efforts to implement evidence-informed interventions may be only partially adopted, reducing their effectiveness (Thürlimann et al., 2022). For instance, Blöndal et al., (2014) found that lectures, workshops, and skills development sessions had no significant impact on HCPs’ attitudes. This underscores the need for improved educational tools and initiatives that promote family involvement in healthcare (Thürlimann et al., 2022). Such initiatives should aim to enhance self-perceived practice skills while fostering confidence and competence in family-systems care. Supervised clinical practice may serve as an effective approach in this regard (Gutiérrez-Alemán et al., 2021).

FSC programs would benefit from being developed and evaluated through implementation research (Gutiérrez-Alemán et al., 2021; Thürlimann et al., 2022). This research should assess outcomes such as acceptability, feasibility, and cost, and examine how these factors influence family health outcomes (Proctor et al., 2023). Given the absence of FSC educational programs in many African countries and their limited inclusion in health training curricula (Abukari & Schmollgruber, 2025; Chironda et al., 2022), it is important that such programs are context-specific, participatory, and responsive to the dynamic healthcare environments.

In our findings, having a FSC approach at the workplace increased perceived practice skills, consistent with previous findings (Barreto et al., 2022). As Ghana currently lacks general guidelines for family involvement (Abukari & Schmollgruber, 2025), it remains unclear how HCPs apply this approach across different units.

Across the units explored, only HCPs stationed in the public health and nutrition unit did not perceive families as a burden. Studies have highlighted variations in family involvement practices across different inpatient care specialists (Cranley et al., 2022; Hetland et al., 2017; Zwicky et al., 2022). Barreto et al. (2022) found that nurses working in an outpatient clinic had a more positive attitude towards families and perceived them as less of a burden than nurses working in an inpatient setting. On the contrary, in this study, outpatient HCPs in health centers or CHPS were more likely to perceive families as a burden than those in the public health and nutrition unit. Such differences have been attributed to the distinct work environments and patient populations encountered in these settings. Lower utilization of specific healthcare services or deficiencies in required skills may influence HCPs’ perception of the role and impact of families in outpatient care (Adusei et al., 2024; Areru et al., 2021). Understanding these dynamics may help promote more positive attitudes towards family involvement across healthcare settings. Future research may qualitatively investigate HCPs’ perceptions of and degree of confidence in FSC implementation.

The sociodemographic characteristics assessed showed no significant influence on HCP attitudes or self-perceived skills. Similarly, Zwicky et al. (2022) found no significant influence of education level, gender, and experience of previous illness on HCPs’ attitudes and skills, while others reported associations (Cranley et al., 2022; Hsiao & Tsai, 2015; Luttik et al., 2017; Rahmqvist Linnarsson et al., 2015). This discrepancy is likely to be caused by contextual or cultural differences, varying sample sizes and in some instances, a lack of statistical power to detect differences (Cranley et al., 2022; Luttik et al., 2017).

### Strengths and Limitations

One notable strength of this study is the comprehensive inclusion of a broad range of HCPs. This inclusive approach broadens the range of perspectives, enriching the depth of the findings and ensuring greater relevance and applicability across various healthcare settings and disciplines.

The limited number of HCPs in each group negatively affects rigorous statistical analysis and generalizability of the findings. Consequently, a subgroup analysis among different HCPs, such as nurses, midwives, or physicians, was not performed, limiting the ability to assess the nuanced effect of HCPs’ characteristics on family involvement. The extent to which professional backgrounds, such as nursing, midwifery, and medicine, influence perceived practice skills and attitudes remains unclear. The role of well-trained (neonatal) nurses in caring for small and sick newborns is critical (Murless-Collins et al., 2025). We acknowledge the limitation that the participants were not exclusively nurses. However, this broader perspective, including diverse teams caring for small and sick newborns across the perinatal care continuum, displays the reality. Future research may focus on nurse-only samples across multiple sites to explore nursing-specific insights more deeply.

### Implication for Practice

The findings emphasize the importance of implementing FSC education programs that consider HCPs’ characteristics and experiences across the perinatal care continuum. Institutions need to be aware of these influential factors and adapt training for nurses, midwives, and medical staff accordingly. Nurses and midwives in leadership positions can promote FSC improvement by integrating FSC guidelines into practice throughout all stages of the perinatal care continuum.

Several factors may hinder the execution of FSC programs, including poor teamwork, limited knowledge sources, and unfavorable work environments such as a lack of privacy (Ndwiga et al., 2022; Schuler et al., 2025). Future research could explore potential mediating factors, such as institutional support, workplace policies, culture, and educational initiatives, to better understand the complex relationships between demographic characteristics and HCPs’ attitudes.

Qualitative research is also needed to capture HCPs’ understanding of how their experiences influence their attitudes and practice skills. Moreover, a qualitative case study should explore the perceived value and applicability of FSC training by participants, specifically nurses and midwives, given that they constitute the largest healthcare group.

LMICs face challenges in implementing FSC programs, a problem exacerbated by global health crises. Engaging diverse stakeholders, including political leaders, organizations, civil society, the private sector, and communities, is crucial for expediting funding and implementation (Agravat et al., 2023; Murless-Collins et al., 2025). Implementation research may leverage alternative funding sources.

Policy reforms in healthcare and training institutions are essential to sustainably integrate FSC by updating professional standards, incorporating pre-graduate and interdisciplinary training, and supporting collaborative, family systemic practices.

## Conclusion

Throughout the continuum of care for small and sick newborns, from antenatal to postnatal home care, we observed that family involvement in the care is often perceived as burdensome. Only one of six care levels, the public health and nutrition unit is perceived otherwise. Transforming attitudes towards FSC at all levels, throughout the continuum from hospital to home, should be targeted to improve the overall quality of newborn care. Prior education in FSC, as well as exposure to FSC at the workplace, influenced perceived practice skills and attitudes toward family involvement in care for small and sick newborns. These findings, together with a significant association between self-perceived practice skills and attitudes, suggest that capacity building in FSC positively influences HCPs’ attitudes and potentially enhances quality of care. Institutional support and education are critical in shaping HCPs’ abilities to engage effectively with families of small and sick newborns across the perinatal care continuum.

## Supplemental Material

sj-docx-1-son-10.1177_23779608251376225 - Supplemental material for How Healthcare Provider Characteristics Affect Their Attitudes and Skills in Involving Families in Caring for Small and Sick Newborns Throughout the Perinatal Period Supplemental material, sj-docx-1-son-10.1177_23779608251376225 for How Healthcare Provider Characteristics Affect Their Attitudes and Skills in Involving Families in Caring for Small and Sick Newborns Throughout the Perinatal Period by Christina Schuler, Alondra Ramos, George Edward Ntow, Riccardo E. Pfister and Faith Agbozo in SAGE Open Nursing
