# Supplementary material for: How Healthcare Provider Characteristics Affect Their Attitudes and Skills in Involving Families in Caring for Small and Sick Newborns Throughout the Perinatal Period
Source: SAGE Open Nurs. 2025 Sep 5;11:23779608251376225. doi: 10.1177/23779608251376225 (PMC12413522; doi:10.1177/23779608251376225)
Supplement: sj-docx-1-son-10.1177_23779608251376225 - Supplemental material for How Healthcare Provider Characteristics Affect Their Attitudes and Skills in Involving Families in Caring for Small and Sick Newborns Throughout the Perinatal Period [file sj-docx-1-son-10.1177_23779608251376225.docx]

## **Supplementary**

**Authors:**

**Christina Schuler^1,2^*, Alondra Ramos^3^, George E. Ntow^4^, Riccardo E. Pfister^1,5,^ Faith Agbozo^6^**

*Corresponding author: Christina Schuler, PhD Student, MScN, MAS International Health, RN, Chemin des mines, 9, CH-1202 Geneva, Switzerland, Phone +41 58 934 61 35, [christina.schuler@etu.unige.ch,](mailto:christina.schuler@etu.unige.ch) [ORCID: 0000-0001-7839-5987](https://orcid.org/my-orcid?orcid=0000-0001-7839-5987)

**Supplementary 1. Multiple Linear Regression model showing the Factors influencing healthcare providers’ perception of families as conversational partners in care (Fam-CP)**

|  | | **Coefficient** | **Standard Error** | | **p-value** | **95% Confidence Interval** | |
| --- | --- | --- | --- | --- | --- | --- | --- |
| **Age** | | .049 | .13 | | .71 | [-.21, .306] | |
| **Gender (ref: Male)** | | -2.53 | 1.28 | | **.05 | [-5.06, 0] | |
| **Educational level (ref: diploma)** | |  |  | |  |  | |
| Certificate | | -1.68 | 1.21 | | .17 | [-4.08, .72] | |
| Bachelor | | -2.30 | 1.51 | | .13 | [-5.30, .69] | |
| Master | | -.84 | 3.63 | | .82 | [-8.03, 6.35] | |
| Other | | -2.45 | 3.47 | | .48 | [-9.31, 4.42] | |
| **Healthcare unit (ref: CHPS**)** | |  |  | |  |  | |
| Antenatal care unit | | 1.88 | 2.47 | | .45 | [-3.00, 6.75] | |
| Labor ward | | 2.36 | 2.13 | | .27 | [-1.83, 6.55] | |
| Neonatal intensive care unit | | 3.25 | 1.88 | | *.09 | [-.48, 6.98] | |
| Postnatal care unit | | -.36 | 2.29 | | .88 | [-4.89, 4.18] | |
| Public health and nutrition unit | | 2.06 | 2.33 | | .38 | [-2.55, 6.67] | |
| Health center | | 1.54 | 1.39 | | .27 | [-1.22, 4.29] | |
| **Healthcare provider skills (FNPS)*** | | -.21 | .07 | | ***0 | [-.34, -.08] | |
| **Prior education in family systems care (ref: No)** | | 1.18 | .99 | | .24 | [-.78, 3.14] | |
| **Family systems care approach at workplace (ref: No)** | | .46 | 1.02 | | .65 | [-1.56, 2.49] | |
| **Work experience in the unit (years)** | | -.07 | .17 | | .67 | [-.42, .27] | |
| **Previous illness (ref: No)** | |  |  | |  |  | |
| Yes | | 1.19 | 1.19 | | .32 | [-1.17, 3.55] | |
| Constant | | 32.58 | 5.06 | | ***0 | [22.56, 42.61] | |
| Mean dependent variable | 29.06 | | | SD dependent variables | | | 5.78 |
| R-squared | 0.22 | | | Number of observations | | | 141 |
| F-test | 1.98 | | | Prob > F | | | 0.018 |
| Akaike crit. (AIC) | 895.67 | | | Bayesian crit. (BIC) | | | 948.75 |
| **** p<.01, ** p<.05, * p<.1* | | | | | | | |

*Dependent variable: Subscale: Family as a Conversational Partner (Fam-CP)*

**FNPS (Family Nursing Practice Scale): Total score ranging from 50 = low skill to 10 = high skill.
**CHPS (Community-based Health Planning and Services)*

**Supplementary 2. Multiple Linear Regression model showing how the openness of healthcare provider towards Families as a their own Resource (Fam-OR) is affected by the socio-demographic characteristics, professional experience and skills of the healthcare providers**

|  | | **Coefficient** | | **Standard Error** | **p-value** | | **95% Confident Interval** |
| --- | --- | --- | --- | --- | --- | --- | --- |
| **Age** | | .014 | | .06 | .82 | | [-.11, .14] |
| **Gender (ref: Male)** | | -1.13 | | .61 | *.06 | | [-2.34, .07] |
| **Educational level (ref: diploma)** | |  | |  |  | |  |
| Certificate | | -.76 | | .58 | .19 | | [-1.9, .38] |
| Bachelor | | -.68 | | .72 | .34 | | [-2.10, .74] |
| Master | | -.26 | | 1.72 | .88 | | [-3.67, 3.15] |
| Other | | -1.30 | | 1.65 | .43 | | [-4.55, 1.96] |
| **Healthcare Unit (ref: CHPS)** | |  | |  |  | |  |
| Antenatal care unit | | .65 | | 1.2 | .58 | | [-1.67, 2.97] |
| Labor ward | | -.35 | | 1.01 | .73 | | [-2.34, 1.64] |
| Neonatal intensive care unit | | .98 | | .89 | .28 | | [-.80, 2.75] |
| Postnatal care unit | | -1.32 | | 1.09 | .23 | | [-3.47, .84] |
| Public health and nutrition unit | | .46 | | 1.11 | .68 | | [-1.73, 2.65] |
| Health center | | 1.22 | | .66 | *.07 | | [-.09, 2.53] |
| **Healthcare provider skills (FNPS)*** | | -.03 | | .03 | .43 | | [-.09, .04] |
| **Prior education in family systems care (ref: No)** | | .96 | | .47 | **.04 | | [.03, 1.89] |
| **Family systems care approach at workplace (ref: No)** | | .71 | | .49 | .15 | | [-.26, 1.67] |
| **Work experience in the unit (years)** | | .10 | | .08 | .22 | | [-.06, .27] |
| **Previous illness (ref: No)** | |  | |  |  | |  |
| Yes | | .30 | | .57 | .60 | | [-.82, 1.42] |
| Constant | | 13.37 | | 2.40 | ***0 | | [8.61, 18.12] |
| Mean dependent variable | 14.87 | | SD dependent variable | | | 2.74 | |
| R-squared | 0.22 | | Number of observations | | | 141 | |
| F-test | 1.99 | | Prob > F | | | 0.02 | |
| Akaike crit. (AIC) | 685.54 | | Bayesian crit. (BIC) | | | 738.62 | |
| **** p<.01, ** p<.05, * p<.1* | | | | | | | |

*Dependent variable: Family as Own Resource (Fam-OR) (FINC-NA subscale)*

*^*^FNPS (Family Nursing Practice Scale): Total score ranging from 50 = low skill to 10 = high skill.
**CHPS (Community-based Health Planning and Services)*

**Supplementary 3. Multiple Linear Regression model showing how much the attitudes towards family involvement are affected by the socio-demographic characteristics, professional experience, and skills of the healthcare providers(FINC-NA Total Scale)**

|  | | **Coefficient** | | **Standard Error** | **p-value** | | **95% Confidence Interval** | | |
| --- | --- | --- | --- | --- | --- | --- | --- | --- | --- |
| **Age** | | .17 | | .32 | .60 | | [-.47, .81] | | |
| **Gender (ref: Male)** | | -5.83 | | 3.19 | *.07 | | [-12.16, .49] | | |
| **Educational level (ref: diploma)** | |  | |  |  | |  | | |
| Bachelor | | -5.68 | | 3.78 | .14 | | [-13.16, 1.80] | | |
| Certificate | | -3.78 | | 3.03 | .21 | | [-9.7, 2.21] | | |
| Master | | 2.67 | | 9.07 | .77 | | [-15.28, 20.62] | | |
| Other | | -2.13 | | 8.66 | .81 | | [-19.28, 15.02] | | |
| **Care unit (ref: CHPS)**^b^ | |  | |  |  | |  | | |
| Antenatal care unit | | 2.42 | | 6.16 | .70 | | [-9.77, 14.60] | | |
| Labor ward | | 1.48 | | 5.29 | .78 | | [-8.99, 11.96] | | |
| Neonatal intensive care unit | | 6.35 | | 4.71 | .18 | | [-2.97, 15.66] | | |
| Postnatal care unit | | -1.26 | | 5.72 | .83 | | [-12.59, 10.06] | | |
| Public health and nutrition unit | | 6.92 | | 5.82 | .24 | | [-4.60, 18.44] | | |
| Health center | | 4.85 | | 3.48 | .17 | | [-2.04, 11.74] | | |
| **Healthcare provider skills (FNPS)^a^** | | -.39 | | .16 | **.02 | | [-.71, -.06] | | |
| **Prior education in family systems care (ref: No)** | | 1.84 | | 2.47 | .46 | | [-3.06, 6.73] | | |
| **Family systems care approach at workplace (ref: No)** | | 1.98 | | 2.56 | .44 | | [-3.08, 7.05] | | |
| **Work experience in the unit (years)** | | .072 | | .44 | .87 | | [-.79, .93] | | |
| **Previous illness (ref: No)** | |  | |  |  | |  | | |
| Yes | | 2.61 | | 2.98 | .38 | | [-3.28, 8.50] | | |
| Constant | | 94.32 | | 12.65 | ***0 | | [69.29, 119.35] | | |
| Mean dependent variable | 91.42 | | SD dependent variable | | | 14.26 | | | |
| R-squared | 0.20 | | Number of observations | | | 141 | | | |
| F-test | 1.76 | | Prob > F | | | 0.04 | |  |  |
| Akaike crit. (AIC) | 1153.79 | | Bayesian crit. (BIC) | | | 1206.87 | |  |  |
| **** p<.01, ** p<.05, * p<.1* | | | | | | | |  |  |

*Dependent variable: Family Importance in Nursing Care – Nurses’ Attitudes (FINC-NA total score)*

*^a^FNPS (Family Nursing Practice Scale): Total score ranging from 50 = low skill to 10 = high skill.
^b^CHPS (Community-based Health Planning and Services)*
